# Advances in Integrated Lignin Valorization Pathways for Sustainable Biorefineries

**DOI:** 10.3390/molecules31020380

**Published:** 2026-01-21

**Authors:** Mbuyu Germain Ntunka, Shadana Thakor Vallabh

**Affiliations:** Department of Chemical Engineering, Faculty of Engineering & The Built Environment, Durban University of Technology, Durban 4000, South Africa

**Keywords:** lignin valorization, biorefineries, depolymerization techniques, catalytic systems, process integration, sustainable bioeconomy

## Abstract

Lignin, the most abundant renewable source of aromatic compounds, plays a pivotal role in advancing sustainable biorefineries and reducing dependence on fossil resources. Recent progress in integrated lignin valorization pathways has unlocked opportunities to convert this complex biopolymer into high-value chemicals, materials, and energy carriers, despite its structural heterogeneity and recalcitrance posing major challenges. This review highlights the significant advancements in depolymerization strategies, including catalytic, oxidative, and biological approaches, which are reinforced by innovations in catalyst design and reaction engineering that enhance selectivity and efficiency. It also discusses emerging technologies, such as hybrid chemo-enzymatic systems, solvent fractionation, and continuous-flow reactors, for their potential to improve scalability and sustainability. Furthermore, this review examines the integration of lignin valorization with upstream pretreatment and downstream recovery, emphasizing process intensification, co-product synergy, and techno-economic optimization to achieve commercial viability. Despite these developments, critical gaps remain in understanding the molecular complexity of lignin, developing universally applicable catalytic systems, and optimizing economic and environmental performance. To guide future research, it poses two key questions: how to design catalysts for selective depolymerization across diverse lignin sources, and how to configure biorefineries for maximum lignin utilization while ensuring sustainability? Addressing these challenges will be essential for lignin’s role in next-generation biorefineries and a circular bioeconomy.

## 1. Introduction

**Figure 1:**
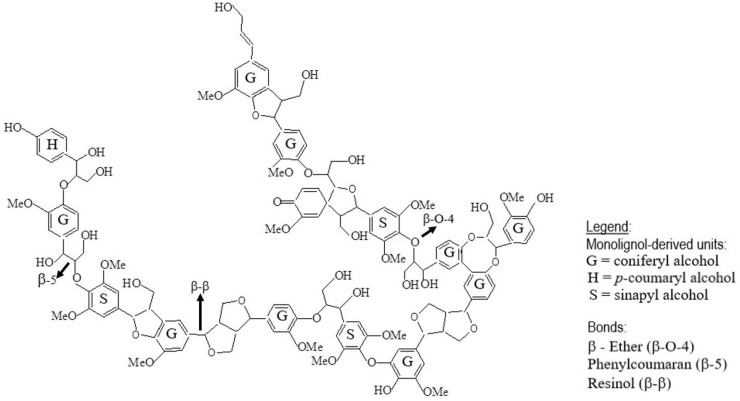
The complex and irregular architecture of lignin, comprising H, G, and S units linked through diverse C–C and C–O bonds (notably the β-O-4 ether bond), underpins the challenge of efficient depolymerization and selective valorization.

Lignin, the most abundant renewable aromatic biopolymer on Earth, is a vast and underutilized resource in the biorefinery landscape [1,2]. Unlike carbohydrates, whose valorization has driven biomass valorization for decades, lignin’s intrinsic heterogeneity, rooted in variations across plant species, tissues, and developmental stages, and its dependence on isolation and processing methods present both formidable challenges and compelling opportunities for integrated biorefinery design [3,4,5]. This heterogeneity is rooted in lignin’s underlying molecular architecture: a complex, irregular polymer composed of phenylpropane units (H, G, S) connected through diverse inter-unit linkages, most notably the β-O-4 ether bond, which critically influences its depolymerization behavior [6]. The coexistence of multiple C–O and C–C bonds contributes to lignin’s remarkable structural diversity and chemical recalcitrance [7]. To visualize this inherent complexity, Figure 1 provides a detailed depiction of lignin’s heterogeneous architecture, highlighting the distribution of monolignols, predominant linkages, and the irregular connectivity patterns characteristic of native lignin [8,9,10].

The structural complexity of lignin is not a fixed attribute; it reflects the source plant lineage, cell wall architecture, and the chemical/enzymatic steps used to liberate and recover lignin [11,12,13]. This source and method-dependent heterogeneity directly impacts processing consistency, product selectivity, and overall biorefinery economics, underscoring the need for robust and adaptable valorization strategies [14,15].

A central message of this review is that lignin’s performance in downstream valorization is governed by its structural fingerprint, which is determined by both its botanical origin and the isolation/pretreatment pathway [16,17]. Recognizing and managing this variability is crucial for achieving reproducible catalytic, oxidative, and biological depolymerization outcomes, as well as for ensuring scalable, industrially relevant processes.

Valuing lignin is not a peripheral add-on but a core pillar of truly integrated biorefineries. By converting lignin into high-value chemicals, polymers, and materials, biorefineries can close material loops, reduce waste, and enhance process intensification. This perspective embodies circular bioeconomy principles, keeping carbon in productive cycles, recovering nutrients wherever possible, and minimizing losses through efficient valorization of all biomass fractions [18,19].

Recent advances in depolymerization and lignin integration strategies are enhancing the profitability of biorefinery while reducing environmental impacts [20,21,22]. However, current industrial processes often burn lignin for energy rather than valorizing it into higher-value products [23,24], despite it representing the largest natural reservoir of aromatic compounds, with over 100 million tons generated annually by various industries and approximately 225 million tons extracted, most incinerated as low-value fuel [17,25].

Lignin valorization is a central pillar for truly integrated biorefineries, enabling them to close material loops, minimize waste, and enhance process intensification while embodying the circular bioeconomy principles of keeping carbon in productive cycles and reducing losses. This underutilization contrasts sharply with its potential as a sustainable source for fuels, chemicals, and materials, underscoring the imperative for advanced valorization strategies [5,23].

Lignin valorization directly supports global sustainability goals, including UN Sustainable Development Goal (SDG) 9, SDG 12, and SDG 15, by transforming this abundant resource from low-value residue into sustainable feedstocks, thereby maximizing resource utilization, minimizing waste, and reducing the carbon footprint.

Several studies have highlighted innovative processes, including catalytic, oxidative, and biological treatments [26,27]. For instance, integrating hydrothermal liquefaction with selective hydrodeoxygenation converts unhydrolyzed solids into value-added biolubricants, reducing the minimum ethanol selling price in corn stover-based biorefineries. Holistic approaches that employ Life Cycle Assessment (LCA) and Circular Bioeconomy principles maximize environmental benefits. Moreover, integrated designs, such as the “Plug-In Processes of Lignin” [23], couple lignin solubilization, conditioning, and fermentation with conventional biorefinery pretreatment methods, thereby overcoming the “lignin-first” versus “carbohydrate-first” dilemma. This paradigm shift enables the simultaneous deconstruction and valorization of all major lignocellulosic biomass components, enhancing biorefinery sustainability and economic viability while facilitating a waste-free system that optimizes the carbon cycle across lignin synthesis, valorization, and product application to foster carbon neutrality [17,24].

This review provides a critical and forward-looking examination of the most advanced lignin modification strategies, highlighting the transformative potential of chemical, biological, amination, acetylation, polymer grafting, and depolymerization approaches for accelerating the shift toward truly sustainable biorefinery operations. Building on this foundation, we demonstrate how recent breakthroughs in catalytic, oxidative, biological, and hybrid depolymerization pathways are converging to enable fully integrated lignin valorization. By emphasizing technical advances, process synergies, sustainability metrics, and economic feasibility, we deliver a comprehensive and persuasive assessment of the challenges, emerging opportunities, and research gaps that will define the next generation of circular biorefineries. Ultimately, we outline strategic, high-impact directions designed to unlock the full value of lignin and position it as a cornerstone resource in future bio-based economies.

## 2. Advances in Depolymerization Techniques

The conversion of lignin into useful products hinges on its depolymerization into smaller molecular fragments. Recent technological advancements emphasize high selectivity, enhanced yields, and compatibility with integrated process schemes. These can be grouped into three primary categories: catalytic approaches, oxidative and chemical depolymerization methods, and emerging biological or hybrid techniques [5,28].

### 2.1. Catalytic Approaches: Hydrothermal Liquefaction (HTL) and Hydrodeoxygenation (HDO)

One of the most promising catalytic strategies for converting lignin or lignin-rich residues into valuable aromatic compounds is HTL followed by HDO [29]. HTL employs subcritical water under elevated pressures and temperatures to dissolve and depolymerize lignin [30]. For instance, the use of a continuous-flow reactor with an induction-heating system has been shown to achieve high efficiencies and rapid heat transfer rates [31]. Specifically, operating the HTL reactor at 320 °C and 115 bar for about one minute, with a controlled amount of phenol as a capping agent and K_2_CO_3_ as a catalyst, maximizes the yield of phenolic monomers by limiting undesirable repolymerization reactions [32].

HTL converts lignin and lignocellulosic feedstocks in subcritical water into bio-crudes and complex phenolic-rich oil. At the same time, HDO upgrades those products toward more stable, reduced, and gas- or liquid-phase fuels and chemicals [33]. The mechanisms in HTL/HDO are governed by the aqueous-phase environment, pressure, temperature, and the presence of a catalyst, as well as the lignin’s structure and pretreatment history [34].

#### 2.1.1. Mechanisms for HTL

The HTL mechanism includes the following:

- Solvolysis and bond cleavage in subcritical water: Elevated temperatures (typically 250–350 °C) and pressures (10–25 MPa) promote the hydrolysis of ether linkages (e.g., β-O-4) and cleavage of C–O–C bonds, aided by water’s autoprotolysis and solvent properties [35].
- Recondensation vs. stabilization: In the absence of stabilizers, reactive intermediates (such as phenolics, ketones, and aldehydes) may repolymerize, forming heavier tar-like products. Water co-solvents and pH control can help steer the process toward more stable products. Moreover, specific capping agents, such as phenol, can effectively prevent repolymerization by reacting with highly reactive lignin fragments, thereby preserving monomeric yields [36].
- Aromatic stabilization and hydrotreatment: Under HTL conditions, in situ hydrogen donation from water or co-solvents, as well as possible hydrogen transfer from the feed, can lead to partial deoxygenation and the formation of more condensed aromatics, depending on temperature, time, and residence time. Crucially, controlling pH is vital for optimal depolymerization during HTL, as demonstrated by studies showing its importance in processing softwood Kraft lignin in subcritical water with specific co-catalysts and capping agents [37].

#### 2.1.2. Key Catalysts and Process Conditions for HTL

Zeolite- or solid-acid catalysts promote dehydration, fragmentation, and rearrangement reactions within HTL matrices [1]. On the contrary, metallic catalysts, such as those incorporating Ni, Ru, or Pt, facilitate hydrogenolysis and hydrogenation reactions, which are critical for both depolymerization and oxygen removal [38].

Homogeneous/heterogeneous metal catalysts (e.g., Ni, Ru, Pd, Ni-based, or supported noble metals) facilitate hydrogen transfer and depolymerization while suppressing char formation when paired with appropriate solvents and hydrogen donors [39]. Further advancements involve integrating these catalytic approaches with other bio-refinery processes to optimize the valorization of diverse lignin streams [31].

Water-rich systems with co-solvents (such as ethanol and glycols) can enhance lignin solubilization, improve heat transfer, and product distribution; alcohols may also act as hydrogen donors [17]. Specifically, the high ionization of water at elevated temperatures, leading to H^+^ and OH^-^ ions, acts as a homogeneous acid-base catalyst for hydrolysis and depolymerization of lignin through selective cleavage of β-O-4 linkages [40,41]. Additionally, the catalytic system plays a critical role in HTL, with various catalysts such as K_2_CO_3_, Na^+^, and Ca^2+^, being employed to facilitate bond scission and modify reaction pathways, thereby mitigating coke formation and enhancing liquid yield [42]. These catalytic systems contribute to the production of various phenolic molecules, including catechol, phenol, and cresols, which are important intermediates for the pharmaceutical and chemical industries [43].

Following HTL, the resulting biocrude, a rich mixture of phenolic compounds, undergoes a two-step hydrodeoxygenation process. In the first step, recalcitrant aromatic rings are hydrogenated over a Pd/C catalyst under controlled conditions, saturating these rings [44]. This step is critical as it stabilizes reactive intermediates while preserving the aromatic backbone. In the subsequent dehydration/dehydrogenation stage, typically conducted in a fixed-bed reactor, oxygen functionalities are further removed, yielding a stream of aromatic hydrocarbons suitable for selective recovery in downstream applications, such as biolubricant production [32].

#### 2.1.3. Mechanisms for HDO

The mechanisms for the HDO process comprise the following:

- Hydrogenolysis of C–O bonds: HDO targets phenolic ethers and other oxygen-bearing linkages, converting phenolic monomers and oligomers into more stable hydrocarbons or hydrocarbon-like bio-oils [45]. This process involves the use of high-pressure hydrogen and heterogeneous catalysts, typically acidic supports with metals like CoMoS, to cleave C–O bonds and remove oxygen as water [46].
- Hydrodeoxygenation steps: Sequential removal of oxygen as water or CO_2_, via catalytic hydrogenation and cleavage of C–O bonds, often involving metal catalysts capable of activating H_2_ and facilitating hydrogen transfer [47]. The precise mechanism often involves initial hydrogenation of aromatic rings, followed by hydrogenolysis of the resulting cyclic ethers, ultimately yielding deoxygenated products [48]. Recent advances, including microwave-assisted pyrolysis and the development of nickel-based zeolite catalysts, have enhanced bio-oil yields and improved hydrodeoxygenation selectivity and stability [5]. Moreover, the catalytic hydrodeoxygenation process shares mechanistic similarities with hydrodesulfurization, utilizing sulfided catalysts to facilitate heteroatom removal through hydrogen consumption [49]. Moreover, the presence of water, either introduced or formed as a product, can significantly influence the HDO pathway by acting as a proton donor/acceptor, reducing the energy barrier for phenolic hydroxyl group removal [50]. The elevated temperatures employed in this second stage facilitate the removal of oxygen from phenolic compounds and other oxygenated species such as ketones, carboxylic acids, and esters [51].

#### 2.1.4. Role of Catalysts and Supports Used for HDO

Ni, Ru, Rh, and Pd on supports (Al_2_O_3_, SiO_2_, ZrO_2_, TiO_2_) are commonly explored; acid modifiers or bifunctional catalysts (acidic + hydrogenation sites) can enhance deoxygenation while maintaining carbon efficiency [47]. This is achieved through a multi-stage process involving initial hydrogenation of aromatic rings and subsequent hydrogenolysis of cyclic ethers [52].

#### 2.1.5. Sustainable Considerations of the HTL and HDO Pathways

Steam reforming, reforming-assisted HTL, or water-gas shift can supply hydrogen in a more integrated, potentially lower-carbon fashion [53]. Furthermore, the development of catalysts capable of operating under milder conditions, such as lower hydrogen pressures, is crucial for reducing process costs and enhancing the overall sustainability of hydrodeoxygenation [54].

HTL matrices can deposit heavy tars on catalysts; robust, regenerable catalysts and proper separation strategies are essential for process viability [33].

Developing catalysts with enhanced coke resistance and regeneration capabilities is therefore paramount to ensuring the long-term economic feasibility of these processes [55].

Early consideration of energy inputs, water use, and product upgrading pathways (e.g., downstream biocrude upgrading vs. direct fuel production) informs the sustainability profile of HTL/HDO routes [55]. Ultimately, a holistic approach that integrates efficient catalyst design, optimized operating conditions, and sustainable hydrogen sourcing is necessary to unlock the full potential of HTL/HDO for biomass valorization.

Furthermore, this catalytic approach not only yields high-value aromatic products but also incorporates heat recovery strategies. For example, the exothermic hydrogenation reaction is coupled with internal heat exchangers to provide heating for subsequent process units, enhancing overall energy efficiency. This integrated design significantly improves economic profitability, as evidenced by a reduced minimum estimated selling price when lignin valorization is coupled with traditional bioethanol production [32,56].

### 2.2. Oxidative and Chemical Depolymerization Strategies

Turning to chemical methods, depolymerization, particularly oxidative approaches, has attracted considerable attention as a means to break down lignin into a range of monomeric compounds, such as vanillin, catechol, and guaiacol [57,58]. These monomers are highly sought after because they serve as key building blocks for the synthesis of bio-based polymers and fine chemicals. Chemical oxidation processes can be carefully tuned to selectively break down specific bonds in lignin while preserving the aromatic core, a challenge given the heterogeneity of lignin.

Mechanistically, lignin depolymerization aims to cleave the dominant interunit linkages (notably β-O-4, β-5, β-β, and 5-5 motifs) to generate lower-molecular-weight aromatics and carbohydrate-derived fragments [57]. The mechanistic pathway and product selectivity are strongly modulated by the oxidation state of lignin, the reaction medium, and the catalyst system [43]. Oxidative depolymerization (ODP) offers a route to relatively milder processing conditions compared with severe hydrocracking, with additional benefits when sustainable oxidants and recyclable catalysts are employed. The performance of ODP is highly sensitive to the lignin’s structural fingerprint, which is in turn shaped by the plant source and the isolation/pretreatment method [17,24].

In addition to oxidative methods, ionic liquids (ILs) have emerged as potent solvents and catalysts for lignin dissolution and depolymerization [59,60]. Although they raise specific toxicity concerns, ILs efficiently disrupt lignin’s highly cross-linked structure. Moreover, they enhance selectivity toward desired monomers while enabling closed-loop recycling, thereby reducing reliance on fossil-based inputs. Overall, this chemical depolymerization approach is particularly beneficial for establishing a circular economy, as recovered monomers can be repolymerized into high-value products without any degradation in quality [22].

Emerging research has also focused on optimizing reaction parameters, such as temperature, oxygen concentration, and catalyst loading, to achieve high yields and selectivity, with the goal of operating under milder conditions that minimize side reactions [61]. This gradual shift toward low-energy and environmentally benign chemical processes reflects the growing need to align lignin valorization with sustainability goals.

### 2.3. Biological and Hybrid Depolymerization Methods

Complementing these chemical strategies, biological approaches involving enzymes and microbial conversion processes offer an attractive, sustainable alternative to conventional methods. Enzymatic degradation, for example, can achieve selective cleavage of specific bonds in lignin under ambient conditions, albeit at generally lower reaction rates [59,62]. Recent studies have explored the use of enzymes to initiate lignin depolymerization, which is subsequently enhanced by mild chemical treatments [63]. One such strategy involves the integration of solubilization and enzymatic fermentation within the so-called Plug-In Process of Lignin (PIPOL), which solubilizes lignin to produce lower molecular weight fractions with increased hydrophilicity, thereby enhancing their bioavailability for microbial fermentation [23].

PIPOL represents a fusion of chemical pretreatment with biological conversion. By incorporating a solubilization stage immediately after conventional biomass pretreatment, it facilitates the conditioning and microbial assimilation of lignin. The resulting process yields valuable bioproducts, such as polyhydroxyalkanoates, while simultaneously improving the enzymatic hydrolysis of the carbohydrate fractions in the biomass. This synergy between lignin valorization and carbohydrate conversion can significantly enhance the overall efficiency and sustainability of biorefineries, addressing the longstanding debate over whether to prioritize lignin-first or carbohydrate-first processing routes [23].

Although biological depolymerization methods are inherently mild and selective, they require extensive optimization to account for the variable structure of lignin [17,64]. Therefore, the development of robust enzymatic cocktails and microbial strains, combined with supportive chemical conditioning methods, is key to unlocking the full potential of biologically based lignin valorization. Microbial strains, for instance, can be genetically engineered to convert lignin-derived monomers into valuable chemicals such as vanillin or muconic acid, which can then be directly marketed or transformed into polymer precursors or fuels [24].

### 2.4. Catalyst Design Principles and Approaches

Building on the biological approaches, catalyst design approaches represent a cornerstone for advancing lignin valorization through targeted catalysts. This critical review examined two complementary catalytic approaches for lignin valorization: heterogeneous chemical catalysis using bimetallic catalysts on porous supports [65] and enzymatic catalysis using multi-copper oxidative laccases [66]. Regarding catalyst design principles, the evidence suggests that strategic pairing of catalyst metals, supports, and solvents represents a fundamental approach for achieving selective lignin conversion through heterogeneous catalysis [65]. In contrast, laccase-based systems require enzyme characterization and optimization to control selectivity and efficiency [66].

Two key studies exemplify these paradigms. Gale et al. [65] emphasize heterogeneous catalysis as a tunable platform for lignin conversion, with particular focus on the strategic pairing of catalyst metals, supports, and solvents as fundamental design principles. The use of bimetallic catalysts on porous supports has emerged as a key strategy for achieving the selective conversion of lignin. This approach leverages the tunable nature of heterogeneous catalysis to control selectivity and efficiency.

In contrast, Curran et al. [66] focus on laccases as biocatalysts for lignin valorization. These multi-copper green oxidative enzymes catalyze bond breaking in lignin to produce smaller oligomers. Consequently, the enzymatic approach offers an alternative mechanism for lignin depolymerization, with the review assessing laccase characterization and optimization as factors controlling selectivity and efficiency. Table 1 provides a comparative summary of various lignin modification strategies, emphasizing their potential for integration into existing biorefinery schemes. The economic viability of these strategies, however, often hinges on factors such as enzyme cost and recyclability [67].

This comparative analysis highlights the need to tailor catalytic strategies to specific valorization objectives, whether favoring robust chemical transformations or milder, selective biological pathways. Notably, neither review details the reaction conditions (e.g., temperature, pressure, pH, or solvent systems) nor lignin feedstock characteristics (e.g., source species, isolation methods, or structural parameters) from the available studies. Moreover, further advancements in oxidative depolymerization use heterogeneous catalysts to upgrade lignin into valuable aromatic monomers, thereby bolstering biorefinery sustainability [58].

## 3. Chemical Functionalization of Lignin Through Amination Processes

Lignin is a heterogeneous aromatic biopolymer whose phenylpropane units and side chains provide sites for nucleophilic/electrophilic functionalization, which can install nitrogen functionalities and alter solubility, reactivity, and interfacial behavior. Amination aims to create more value by (i) keeping lignin pieces stable during fractionation/depulping, (ii) adding reactive primary and secondary amines to allow for more polymer chemistry, and (iii) making small molecules that contain nitrogen during depolymerization. These molecules broaden the range of products that biorefineries can offer. The chemical literature indicates that amination can occur during pretreatment (in situ), by grafting (via Schiff base formation or amide synthesis), or through Mannich-type alkylation to produce cationic or multifunctional lignins, which enable downstream applications such as adhesives, emulsifiers, and monomeric aromatic amines [68,69,70,71].

### 3.1. Amination Chemistries

Reductive amination is another method for achieving amination, which involves introducing an amine group through a reaction with an aldehyde or ketone. These various amination chemistries and mechanisms provide a diverse range of options for modifying lignin in biorefineries.

- Amination via activated heterocycles (oxazolidinone/oxazoline) installs amide linkages that are subsequently hydrolyzed to yield free primary amines; this two-step route was explicitly designed to avoid toxic reagents and to provide selective, reactive primary amines on lignin chains [70].
- Amines can react during pretreatment to form imines/Schiff bases and amide linkages with lignin side chains; these transformations can both stabilize reactive lignin fragments against condensation and create new functional groups for downstream uses [69].
- Mannich chemistry (formaldehyde + amine + activated aromatic site) introduces cationic tertiary/aminoalkyl groups suitable for surfactants and adhesives; reaction performance depends strongly on pH and stoichiometry [71].
- Under depolymerizing/hydrogenolysis conditions, nucleophilic amines can trap lignin side-chain fragments to give N-containing small molecules (e.g., pyridine bases or phenolic amines) when tandem fractionation and amination are combined [68].
- Microwave-assisted amination can facilitate nucleophilic attack on C–C bonds in model compounds, resulting in amide formation and heteroatom doping that modifies polarity and adsorption behavior [72].

### 3.2. Applications of Amination Processes and Biorefinery Integration

Reports demonstrate aminated lignins functioning as adhesives, surfactants, lubricating additives, enzyme supports, and precursors to aromatic amines/pyridine bases. Several studies demonstrate routes to integrate amination into pretreatment or depolymerization to improve overall biorefinery valorization.

#### 3.2.1. Adhesives and Wood Panels

Amination and crosslinking of acetone-fractionated hardwood Kraft lignin with ethylenediamine (EDA) or diethylenetriamine (DEA), followed by aldehyde crosslinkers, produced adhesives meeting Korean plywood standards; FT-IR, 13C/15N NMR and XPS confirmed amide/imine formation [73].

Aminated lignin-Cu nanoparticles grafted with amino-terminated hyperbranched polyamide yielded a formaldehyde-free adhesive with bond strength 1.51 MPa, debonding work 0.272 J, and wet shear 1.15 MPa, and ~100 day mildew resistance, prepared in water without organic solvents [74].

#### 3.2.2. Surfactants and Emulsifiers

Mannich amination of Kraft lignin with tetraethylene pentamine/formaldehyde at pH 13 produced cationic lignin surfactants that stabilized 60% bitumen emulsions using 0.25–0.75% surfactant; reagent molar ratios (lignin/amine/formaldehyde) between 1/7/7 and 1/28/28 were explored [71].

#### 3.2.3. Lubricant Additives and Tribology

Microwave amination and heteroatom doping with tris(2-hydrazinylethyl)borate grafted N and B groups, achieved via C–C bond cleavage, resulted in higher polarity aminated lignins that improved PEG200 viscosity and reduced wear and friction in tests [72].

#### 3.2.4. Enzyme Immobilization and Flow Biocatalyst Supports

Polyethyleneimine-lignin and other amine/epoxy/aldehyde-functionalized lignins afforded immobilization yields of 64–100% and reversible enzyme capture. A packed-bed reactor retained conversion for 100 cycles, demonstrating operational stability in a lignin-based flow bioprocess [75].

#### 3.2.5. Monomer Production and Chemical Building Blocks

In situ amination during fractionation with aqueous DEA enabled lignin stabilization and, after tandem hydrogenolysis, co-production of monophenolics and pyridine bases in high yield from real lignin, effectively converting cleaved lignin fragments to N-containing small molecules, expanding product portfolios for biorefineries [68].

#### 3.2.6. Flocculation and Water Treatment

EDA-aminated lignins from EDA pretreatment exhibited pH-responsive behavior. They reduced kaolin turbidity by more than 90%, while simultaneously improving carbohydrate yields from the solids fraction (96% glucose, 70% xylose), demonstrating an integrated benefit of pretreatment and valorization [69].

#### 3.2.7. Integration Strategies Shown in the Literature

In situ amination during fractionation or pretreatment using DEA or EDA stabilizes lignin against condensation while simultaneously enabling the coproduction of nitrogen-containing small molecules or macromolecules. This dual functionality enhances overall mass balance and improves downstream process economics [68,69].

Grafting approaches (Schiff base, amination, and Mannich) applied to technical lignins after isolation yield tailored materials, including adhesives, surfactants, and polymer additives. These products can often be processed in water or mild solvents, reducing environmental impact [70,71,74].

Furthermore, integrating amination with catalytic depolymerization or hydrogenolysis can direct product distributions toward high-value nitrogen-containing monomers, such as phenolic amines and pyridine bases, which offer greater value than conventional bulk lignin-derived fuels [68,75].

### 3.3. Sustainability Economics Challenges

Sustainability benefits, such as benign reagents, formaldehyde-free formulations, and co-production opportunities, are well documented, yet economic and technical barriers remain, including feedstock variability, reagent cost/toxicity, and scale-up challenges for selective amination. Benign amination using N-acetyl-2-oxazolidinone or 2-methyl-2-oxazoline avoids conventional toxic chemistries and provides reactive amines for polymer applications [70]. Water-based nanoparticle adhesives similarly reduce toxic emissions and solvent use while maintaining competitive performance and regulatory advantages [74]. In situ amination during fractionation (DEA/EDA) stabilizes lignin and enhances sugar release, improving biorefinery yields and economics [68,69]. Upgrading lignin fragments to aromatic amines or pyridine bases offers a higher value than combustion but requires selective catalysis and controlled depolymerization [68,75].

## 4. Integration Strategies in Lignin Valorization

The successful conversion of lignin into value-added products depends not only on effective depolymerization methods but also on the integration of these processes with upstream pretreatment and downstream recovery techniques. Integrated strategies enhance process efficiencies, enable resource recovery, and improve the economic viability of biorefineries.

### 4.1. Upstream Integration with Pretreatment Technologies

Upstream processes in biorefineries have traditionally focused on extracting fermentable carbohydrates from lignocellulosic biomass via pretreatment. However, these same steps often generate substantial lignin-rich residues. An optimal pretreatment balances cellulose and hemicellulose recovery with effective delignification, while minimizing inhibitors that impede downstream enzymatic or chemical conversions. Widely studied methods include dilute sulfuric acid (DSA), liquid hot water (LHW), steam explosion (SEP), ammonia fibre expansion (AFEX), and sodium hydroxide pretreatment (SHP) [23,76].

Building on these methods, a key innovation couples traditional pretreatments with “plug-in” strategies, such as PIPOL, which target lignin solubilization by lowering its molecular weight and enhancing hydrophilicity [23]. For instance, during LHW or DSA, the solubilized lignin stream can be redirected for catalytic depolymerization or biological conversion, without interfering with the carbohydrate fraction for enzymatic hydrolysis and subsequent ethanol production. This upstream integration improves overall resource utilization and reduces biorefinery waste. Furthermore, advancements in genetic engineering of bioenergy crops are producing lignin feedstocks with tailored properties that facilitate easier recovery and conversion processes, thereby further streamlining upstream integration [53].

Table 2 summarizes the key characteristics of various pretreatment methods and their compatibility with integrated lignin valorization processes. This includes optimizing pretreatment severity to yield lignin with specific structural features desirable for subsequent valorization, thus enabling a holistic approach to biomass utilization [53]. Moreover, ongoing research continues to explore novel “lignin-first” fractionation strategies that selectively depolymerize and stabilize lignin during biomass pretreatment, preventing condensation reactions that typically hinder its downstream valorization and ensuring its suitability for diverse applications [24].

As illustrated in Table 2, this holistic approach highlights the shift towards biorefinery designs that simultaneously optimize carbohydrate processing and facilitate efficient lignin valorization, aligning with the United Nations Sustainable Development Goals (UN SDG) 9 (industry innovation), 12 (responsible consumption and production), and 15 [23,24]. By optimizing pretreatment severity to yield lignin with specific structural features desirable for valorization, biorefineries move toward maximizing resource utilization and minimizing waste.

### 4.2. Downstream Integration and Product Recovery

Complementing upstream pretreatment integration, downstream strategies play an equally critical role in ensuring lignin valorization processes are both technically feasible and economically viable. A compelling example is the production of biolubricants from lignin-derived aromatic compounds. In an integrated biorefinery scheme, the lignin fraction, typically removed from the combustor, is redirected to a catalytic conversion process. This first produces biocrude via HTL and then converts it into high-purity aromatic hydrocarbons via HDO, which are subsequently used to alkylate fatty acid methyl esters (FAMEs) derived from waste cooking oil (WCO), yielding phenyl-branched biolubricants [32].

This downstream approach not only increases the portfolio of bio-based products available from a single feedstock but also provides additional revenue streams that can offset increased capital and variable operating expenses. Heat integration plays a significant role in this process, as the exothermic and endothermic reactions within the HTL, HDO, and alkylation units are interconnected through optimized heat exchangers and distillation columns. For instance, the cooling requirements in the double-step HDO process are utilized to preheat incoming reagents, thereby reducing net energy consumption and improving overall process sustainability.

**Figure 2:**
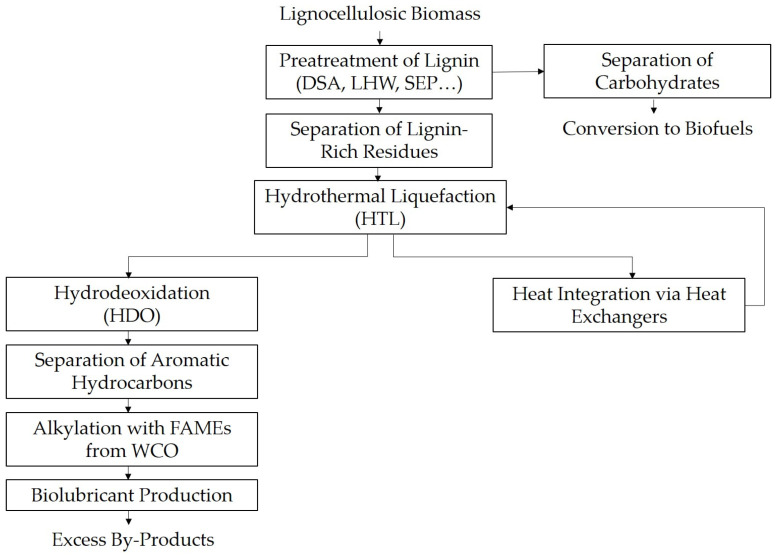
Integrated Downstream Process Flow for Lignin Valorization Leading to Biolubricant Production.

The following flowsheet in Figure 2, based on the work described by Barbera et al. [32], illustrates the downstream integration of lignin valorization within a biorefinery:

### 4.3. Process Intensification and Modular Approaches

In addition to upstream and downstream integrations, process intensification and modular approaches further enhance efficiency. Process intensification involves restructuring processing units to enhance productivity, reduce energy consumption, and minimize the plant’s footprint. For instance, the adoption of continuous-flow reactors in advanced HTL systems exemplifies improved depolymerization performance through rapid heating, more uniform reaction conditions, and simpler heat integration compared to conventional batch systems [32].

Moreover, modular technologies such as PIPOL are gaining traction for their ability to “plug into” existing biorefinery infrastructures without requiring a complete facility overhaul. By sequentially integrating solubilization, conditioning, and fermentation, PIPOL enables efficient conversion of lignin into fermentable intermediates that can directly yield bio-based products or serve as building blocks for further chemical transformations [23]. The modular nature of these processes enables flexible scaling and incremental upgrades, allowing for adjustments based on feedstock availability and market demand.

**Figure 3:**
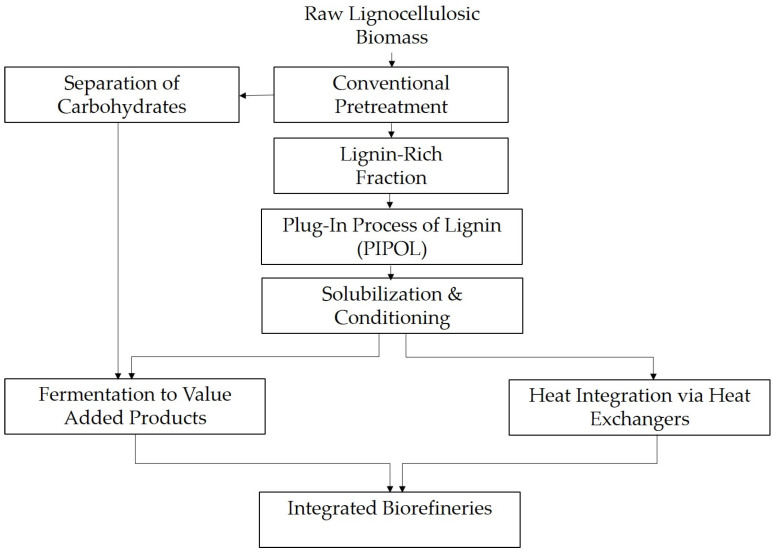
Modular Integration of PIPOL into Existing Biorefinery Systems.

The diagram in Figure 3, based on the work described in Liu et al. [23], provides a schematic representation of a modular integrated lignin valorization system:

Ultimately, this modular approach not only simplifies future scaling but also facilitates rapid process optimization, accelerating the transition to commercially viable integrated lignin valorization pathways. Furthermore, this strategy significantly reduces the economic risk associated with large-scale biorefinery investments by allowing for phased implementation and de-risked technology deployment [77]. Such modularity is particularly beneficial for incorporating nascent catalytic systems, such as bimetallic catalysts and nanostructured supports, which have demonstrated enhanced selectivity and reduced fouling in lignin depolymerization, coupled with machine learning-assisted workflows that bridge the gap between predictive modeling and experimental validation [5].

## 5. Challenges and Research Gaps

Despite the promising advances in lignin valorization, several challenges remain that must be addressed for widespread industrial implementation. These challenges span technical, economic, and environmental domains. Foremost among these is the inherent heterogeneity and complex chemical structure of lignin, which varies significantly between plant species and isolation methods, complicating consistent processing and product development [78,79].

### 5.1. Structural Complexity and Catalyst Deactivation

Lignin is a highly heterogeneous biopolymer, characterized by a diverse array of inter-unit linkages and a large molecular weight distribution. This inherent structural complexity makes it difficult to achieve a uniform and selective depolymerization process [80]. In catalytic processes such as HTL and HDO, the differing reactivity among lignin bonds leads to a broad distribution of products, many of which are unsuitable for downstream applications [81]. Moreover, the harsh reaction conditions required to break robust carbon–oxygen bonds often result in catalyst deactivation, for instance, water present during HDO can poison the catalyst, thereby reducing selectivity toward aromatic hydrocarbons [32,82].

To address these issues, researchers are actively exploring novel catalyst formulations that resist deactivation while promoting higher selectivity under milder conditions [82,83]. Innovative approaches include multi-functional catalysts, supported or bimetallic systems, and the incorporation of green solvents to enhance reactivity and stability [84]. In addition, the high chemical stability and natural complexity of lignin restrict its applications, necessitating efficient and environmentally sound technologies for its degradation into useful bioproducts [28].

### 5.2. Economic and Environmental Trade-Offs

Economic viability is a critical factor in the adoption of lignin valorization technologies. Techno-economic analyses demonstrate that integrating these processes into existing biorefineries can significantly reduce the minimum ethanol selling price, for example, from approximately $0.798/L to $0.697/L [32,85]. However, economic performance remains sensitive to fluctuations in feedstock prices and market prices for value-added products. Sensitivity analyses reveal that variations as small as ±20% in key inputs or products can markedly affect overall feasibility [32].

From an economic standpoint, as highlighted by sensitivity analyses, viability hinges on stable input costs; turning to environmental aspects, lignin valorization offers significant potential to reduce greenhouse gas emissions (GHG) and reliance on fossil-based inputs. However, life cycle assessments reveal that improvements are not uniform across all indicators: while substituting fossil materials with lignin-derived products results in modest reductions in GHG emissions and fossil fuel depletion, other categories, such as ecotoxicity, wastewater treatment, and land use changes, may actually increase [86]. For instance, the generation of wastewater and potential leaching issues during chemical treatments necessitate comprehensive management strategies to reduce adverse environmental impacts.

Table 3 below summarizes key economic and environmental trade-offs in integrated lignin valorization processes.

A comprehensive evaluation across various dimensions of energy, environment, and economy (3E analysis) indicates that integrating lignin valorization, particularly through gasification-syngas fermentation, enhances carbon and energy recovery while providing significant environmental and economic benefits [87]. These benefits are realized through a reduction in the payback period and an increase in net profit, as observed in studies evaluating lignin-based biorefineries [77]. Furthermore, diverting lignin to produce high-value co-products like phenolics can significantly decrease the cost of ethanol production, leading to a minimum selling price of $2.02 per gallon while achieving a substantial 78% reduction in greenhouse gas emissions compared to gasoline [56]. The valorization of lignin into diverse bioproducts, such as 2-pyrone-4,6-dicarboxylic acid from hardwood, presents significant economic potential with high net present values and internal rates of return, largely driven by revenue from lignin-based products [84].

This highlights the necessity for meticulous process design and optimization to mitigate trade-offs and maximize sustainability [85,87]. In this regard, developing novel plug-in processes for lignin conversion is likewise essential to bolster biorefinery competitiveness and advance the circular bioeconomy [23,88]. Ultimately, effective valorization, transforming this abundant biopolymer into sustainable feedstocks, is critical for the economic success of aqueous biorefineries and for aligning with global sustainability goals, such as UN SDGs 9, 12, and 15 [24,89]. For example, integrated lignin valorization improves carbon and energy recovery while enhancing environmental and economic benefits, reducing the minimum ethanol selling price to an estimated 834–873 $/t for various utilization processes [87]. Therefore, continued research is crucial to optimize these processes, minimize energy demands, and enhance the overall sustainability of lignin-based biorefineries [77,85].

### 5.3. Scalability and Sustainability Issues

While laboratory and pilot-scale demonstrations have proven the technical feasibility of various lignin valorization techniques, scaling these processes to commercial levels remains challenging. For instance, pilot systems such as continuous-flow hydrothermal liquefaction reactors have shown promising performance metrics; however, uncertainties persist regarding reactor design, catalyst lifetime, and process integration during the transition from experimental to full-scale operations [32].

In addition, sustainability is another key consideration. Integrated biorefineries must economically justify the additional capital expenditures and variable operating expenditures needed for incorporating new lignin conversion units. For instance, although the integration of biolubricant production increases CAPEX by approximately 63% and OPEX by 330%, the resultant by-product revenues can offset these costs, provided that market conditions remain favorable [32]. Thus, robust process optimization, along with the development of sustainable catalyst technologies and improved feedstock management strategies, is essential to ensure long-term viability.

Furthermore, a comprehensive sustainability assessment must encompass not only techno-economic factors but also environmental and societal impacts. LCA has emerged as a vital tool in this regard, helping to identify critical environmental hotspots—from wastewater treatment to land use change, that need to be addressed in future process designs [86].

A comprehensive understanding of these interconnected factors is imperative for developing economically viable and environmentally sound lignin valorization pathways that can genuinely contribute to a circular and low-carbon economy [90]. Moreover, the ongoing challenges in depolymerizing lignin into simple aromatic molecules necessitate continuous innovation in catalytic processes and biorefinery configurations to enhance cost-competitiveness and expand the range of valuable bio-based products [14].

## 6. Future Perspectives and Directions

The future of integrated lignin valorization lies in a multidisciplinary approach that encompasses advances in catalysis, process intensification, modular integration, and sustainability assessment. Key directions for future research include:

### 6.1. Advanced Catalyst Development

Researchers should prioritize highly selective, deactivation-resistant catalysts operable under milder conditions. Multi-functional and bimetallic systems, supported by green solvents or ionic liquids, promise efficient depolymerization with minimal side reactions. Catalysts enabling lignin conversion to aromatic monomers, particularly via oxidative processes integrable into biorefineries, are crucial [22,32,58]

### 6.2. Hybrid Chemo-Biological Processes

Integrating chemical and biological methods, as in the PIPOL approach, overcomes limitations of individual techniques. Hybrid systems that solubilize and condition lignin before enzymatic conversion promise high yields of fermentable intermediates and tailored monomers. This synergy mitigates harsh chemical conditions while boosting biological specificity and efficiency for high-value biochemicals [21,23].

### 6.3. Modular and Scalable Reactor Designs

Transitioning to continuous processing, exemplified by induction-heated HTL reactors, enables intensification and scalability. Modular designs facilitate integration into biorefineries, supporting incremental improvements and flexible capacities. Advancements like microreactors and oscillatory flow reactors could enhance mass/heat transfer, reaction control, and selectivity [5]. Coupled with advanced process control and real-time monitoring, these will optimize commercial-scale throughput and product quality.

### 6.4. Integrated Process Optimization

Enhanced integration of pretreatment, conversion, and recovery, supported by simulations and energy analyses, will minimize energy use and costs. Heat integration, including recovery from exothermic reactions, must be optimized for low net energy demand [32].

### 6.5. Comprehensive Sustainability and LCA Studies

Comprehensive LCA studies are essential to assess environmental impacts, including GHG emissions, water usage, and land use changes, ensuring alignment with circular bioeconomy principles. Interdisciplinary collaboration among engineers, environmental scientists, and sustainability experts is needed, alongside evaluations of capital and operational expenditures for commercialization [86,88]. This requires collaboration between process engineers, environmental scientists, and sustainability experts.

### 6.6. Economic and Market Analysis

Techno-economic analyses (TEA), including sensitivity studies on feedstock costs, product prices, and parameters, will identify viable configurations. Policy incentives and regulations will support commercial success. Integrating artificial intelligence (AI) and machine learning for catalyst design and pathway optimization will accelerate cost-effective, environmentally benign processes [5].

### 6.7. Enabling Novel Applications of Lignin-Derived Products

Beyond fuels and biolubricants, lignin-derived products offer potential in bioplastics, bio-composites, and advanced materials like bio-leather. Research into novel applications and polymer synthesis with lignin monomers could replace petroleum plastics with renewable, biodegradable alternatives, opening new revenue streams [22].

Converging these directions will evolve next-generation biorefineries that fully exploit lignin’s potential, transforming it from low-value waste into a cornerstone of sustainable bioprocessing for environmental protection, resource conservation, and economic development.

## 7. Conclusions

Integrated lignin valorization represents a transformative opportunity for sustainable biorefineries, offering enhanced feedstock utilization and reduced environmental impact. Advances in depolymerization techniques, from catalytic processes like HTL and HDO to innovative chemical and biological methods, along with integration strategies such as upstream pretreatment coupling and downstream recovery, have demonstrated potential to lower operating costs and improve economic performance through reduced MESP figures.

Nevertheless, lignin’s structural complexity, catalyst deactivation, process scalability, economic fluctuations, and environmental trade-offs, such as wastewater management, pose significant hurdles. Life Cycle Assessments indicate reduced GHG emissions and fossil dependency, but additional measures are needed to address unintended burdens.

*Future research must focus on the following:*

- Developing advanced catalysts with enhanced selectivity and stability.
- Employing hybrid chemo-biological processes, such as PIPOL, for synergistic conversion.
- Designing modular, scalable reactor systems for seamless biorefinery integration.
- Conducting comprehensive LCAs alongside techno-economic analyses to ensure sustainability.

These efforts, underpinned by rigorous techno-economic and environmental analyses, will drive the transition from laboratory-scale innovation to commercially viable and sustainable bioprocessing. In summary, integrated lignin valorization is poised to become a cornerstone of sustainable biorefineries, paving the way for a greener, more circular bioeconomy.

*Key findings:*

- Lignin is a renewable aromatic resource with high potential for value-added conversion.
- Catalytic processes, particularly HTL coupled with HDO, efficiently produce aromatic monomers.
- Chemical oxidation, ionic liquid methods, biological strategies, and hybrid systems, like such as PIPOL, improve solubilization, monomer recovery, and conversion rates.
- Integration with pretreatment and downstream recovery enhances economics and efficiency.
- Challenges from lignin’s complexity, deactivation, scalability, and trade-offs require holistic designs.
- Continued research into chemical and biological depolymerization routes is crucial for sustainable biorefineries, prioritizing minimized energy for solvent separation, optimized reactors to reduce capital costs, and full utilization of lignin oils [21,85].

Table 4 synthesizes primary conclusions on advancements and challenges in lignin valorization, highlighting innovative approaches from genetic crop manipulation to advanced analytics and modeling [23,53].

The concerted progress in catalytic systems, process integration, and sustainability assessments will transform lignin from low-value by-product to bioeconomy cornerstone, enabling seamless biorefinery integration for maximum value, improved economics, and environmental benefits.

In conclusion, the future of sustainable biorefineries depends on the seamless integration of lignin valorization technologies. By leveraging advanced depolymerization techniques and integrating them effectively with upstream and downstream processes, it is possible to extract maximum value from lignocellulosic biomass. This integrated approach promises not only improved economic metrics but also significant environmental benefits, setting the stage for a transition toward a truly circular and sustainable bio-based industry.

## Figures and Tables

**Table 1:** Comparison between the heterogeneous catalysis design approach and the enzymatic design approach.

Design Aspect	Heterogeneous Catalyst Approach	Enzymatic Approach	References
Catalyst type	Bimetallic catalysts on porous supports	Multi-copper oxidative enzymes (laccases)	[65,66]
Key design principles	Pairing of catalyst metals, supports, and solvents	Enzyme characterization and optimization	[65,66]
Mechanism	Not explicitly detailed	Oxidative bond breaking to produce oligomers	[65,66]
Tunability	Highlighted as a tunable platform	Subject to characterization and optimization efforts	[65,66]

**Table 2:** Comparative Analysis of Pretreatment Methods and Their Integration Potential with Lignin Valorization.

Pretreatment Method	Key Features	Integration with Lignin Valorization	Challenges	References
DSA	High delignification; effective hemicellulose solubilization	Compatible with post-solubilization processes (e.g., PIPOL)	Formation of inhibitors	[23]
LHW	No additional chemicals; sustainable approach	Enables lignin solubilization and immediate recycling into HTL or fermentation modules	High energy consumption	[76]
SEP	Rapid process; partial depolymerization of lignin	Requires downstream conditioning for efficient valorization	High solids loading may hinder complete processing	[76]
AFEX	Preserves carbohydrate structure; moderate lignin removal	Lignin “plug-in” processes can address residual fractions	Ammonia recovery and recycling complexity	[76]
SHP	Direct extraction of lignin; high purity for subsequent bioconversion	Provides high-quality lignin for catalytic and enzymatic conversion	Generates caustic effluents requiring treatment	[23]

**Table 3:** Economic and Environmental Trade-offs in Integrated Lignin Valorization Processes.

Parameter	Improvement with Integration	Potential Drawbacks	References
Minimum Energy Selling Price (MESP)	Decrease from $0.798/L to $0.697/L	Sensitive to feedstock and product price fluctuations	[32]
GHG	Reduction due to substitution of fossil inputs	Increased impacts in wastewater handling and land use	[86]
Energy Efficiency	Enhanced through process and heat integration	High electricity demand (e.g., induction heating)	[32]

**Table 4:** Summary Table of Main Findings.

Aspect	Key Advances	Challenges/Concerns	References
Catalytic Depolymerization	HTL at 320 °C, 115 bar; 2-step HDO mechanism	Catalyst deactivation by water; harsh conditions	[32]
Oxidative/Chemical Techniques	Use of ionic liquids for selective oxidation	Toxicity concerns; high energy requirements	[22]
Biological/Hybrid Conversion	PIPOL for solubilization and fermentation	Lower reaction rates; process optimization needed	[23]
Upstream Integration	Coupling with DSA, LHW, SEP, AFEX, and SHP	Formation of inhibitors; energy demands	[23,76]
Downstream Integration	Biolubricant production via aromatic alkylation	High CAPEX/OPEX; feedstock sensitivity	[32]
Economic & Environmental Impact	Reduction in MESP; potential GHG reduction	Sensitivity to market conditions; wastewater issues	[32,86]

## Data Availability

No new data were created or analyzed in this study. Data sharing is not applicable to this article.

## Abbreviations

The following abbreviations are used in this manuscript:

HTL	hydrothermal liquefaction
HDO	hydrodeoxygenation
ODP	oxidative depolymerization
IL	ionic liquids
PIPOL	plug-in process of lignin
DSA	dilute sulfuric acid
LHW	liquid hot water
SEP	steam explosion
AFEX	ammonia fiber expansion
SHP	sodium hydroxide pretreatment
FAME	fatty acid methyl esters
WCO	waste cooking oil
GHG	greenhouse gas
MESP	minimum energy selling price
UN SDG	united nations sustainable development goal
CAPEX	capital expenditure
OPEX	operating expenditure
LCA	life cycle assessment
TEA	techno-economic analysis
AI	artificial intelligence
EDA	ethylenediamine
DEA	diethylamine
